# Computational optimum of recurrent neural circuits at intermediate numbers of nonlinear dendritic branches

**DOI:** 10.1186/1471-2202-14-S1-P273

**Published:** 2013-07-08

**Authors:** David Breuer, Marc Timme, Raoul-Martin Memmesheimer

**Affiliations:** 1 Network Dynamics, Max Planck Institute for Dynamics & Self-Organization, Göttingen, Germany; 2 Bernstein Center for Computational Neuroscience Göttingen, Göttingen, Germany; 3 Fakultät für Physik, Georg-August-Universität Göttingen, Göttingen, Germany; 4 Max Planck Institute of Molecular Plant Physiology, Potsdam-Golm, Germany; 5 Donders Institute, Department for Neuroinformatics, Radboud Universiteit Nijmegen, Nijmegen, Netherlands

## 

**Figure 1:**
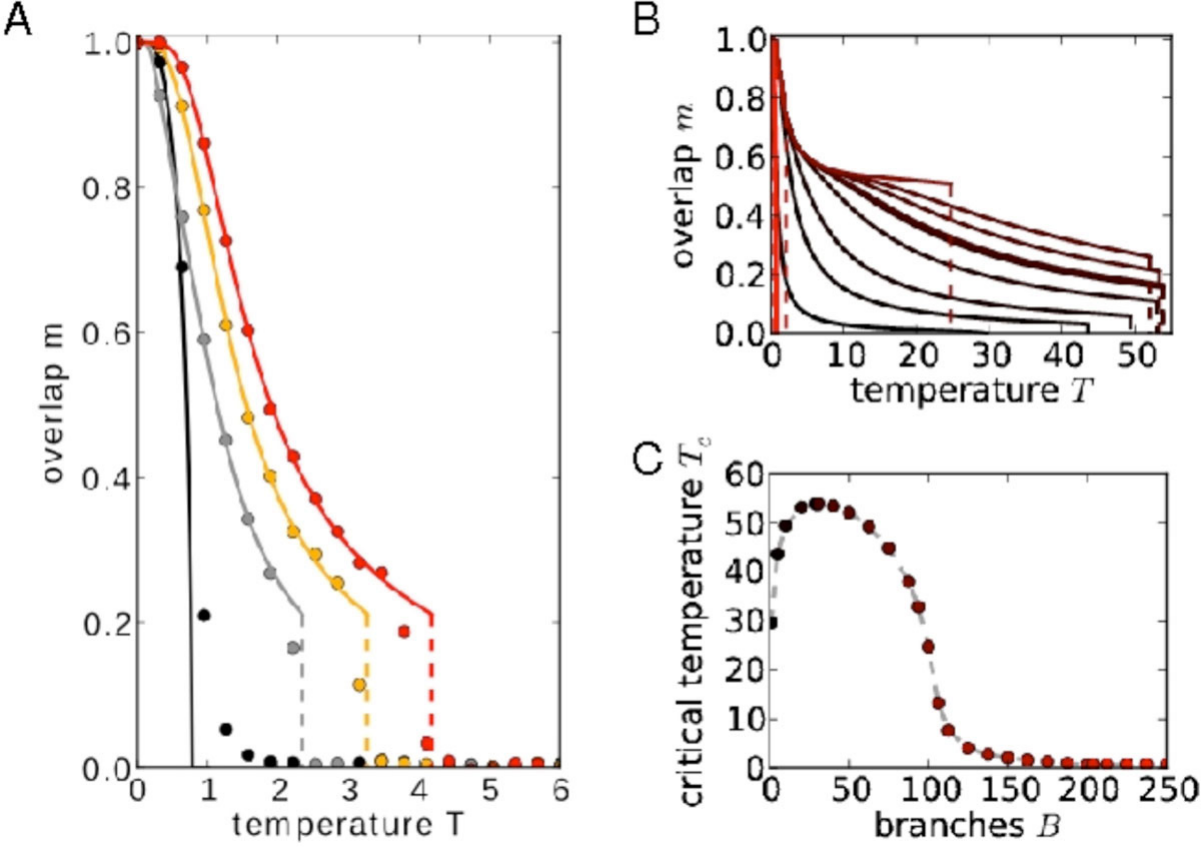
**Nonlinear dendrites increase the robustness of associative memory retrieval against noise, optimally at an intermediate number of branches**. (A) Overlap m of the network state with a retrieval pattern for linear dendrites (black) and increasing strengths of the dendritic nonlinearity (gray, yellow, red). The critical temperature T_C _at which the overlap becomes zero increases with increasing nonlinearity. Analytical and numerical results are given by continuous curves and circles, respectively. (B) Overlap vs. temperature-curves for different numbers of dendritic branches, increasing branch numbers are color-coded from black to red. The critical temperature depends non-monotonically on the number of branches and assumes a maximum at an intermediate value (C).

How neurons process their inputs crucially determines the dynamics of biological and artificial neural networks. Synaptic input is typically considered to be merely transmitted linearly or sublinearly by the dendritic compartments. Yet, single-neuron experiments report pronounced supralinear dendritic summation of sufficiently synchronous and spatially close-by inputs. Here, we study its influence on single neuron responses and the performance of associative memory networks. First, we compute the effect of random input to a neuron incorporating nonlinear dendrites. This approach is independent of the details of the neuronal dynamics. Second, we use those results to study the impact of dendritic nonlinearities on the network dynamics in a Hopfield-type associative memory model, both numerically and analytically. We find that dendritic nonlinearities maintain network convergence and increase the robustness of memory performance against noise (Figure 1A). Interestingly, an intermediate number of dendritic branches is optimal for memory functionality (Figure 1B,C).

